# Theoretical and Experimental Study on the Effect of Selected Parameters in a New Method of Extrusion with a Movable Sleeve

**DOI:** 10.3390/ma15134585

**Published:** 2022-06-29

**Authors:** Grzegorz Winiarski

**Affiliations:** Faculty of Mechanical Engineering, Lublin University of Technology, 36 Nadbystrzycka Str., 20-618 Lublin, Poland; g.winiarski@pollub.pl

**Keywords:** extrusion, hollow part, metal forming, unconventional processes of metal forming, upsetting, flanging

## Abstract

This paper presents a new method for forming hollow flanged products. The method involves extrusion with the use of a sleeve moving in the opposite direction to that of the punch. A tube with a constant hole diameter and two different outside diameters, made of aluminum alloy EN AW 6060 was used as a material. Numerical calculations were performed using Deform 2D/3D. Experiments were conducted on the PYE 160SS hydraulic press equipped with a specially designed device in which the punch is driven by the press slide while the moveable sleeve is driven by two hydraulic servomotors. Both numerical simulations and experiments were conducted under cold forming conditions. The aim of this study was to determine the effect of selected parameters (flange diameter, height of the cavity in the moveable sleeve, and the chamfer angle between the regions with different outside diameters on the workpiece and in the moveable sleeve cavity) on the stability of the extrusion process. Results were then used to undertake detailed comparative analyses of underfill, flange heights, and flange flank inclination angles. Findings of the analyses served as a basis for drawing conclusions regarding the effect of the analyzed parameters on the investigated extrusion process.

## 1. Introduction

Hollow products can be manufactured by metal forming methods. These methods are classified according to different criteria, including, among others, material temperature, tool and billet kinematics, machine type, or billet geometry. Bars and tubes are the most widely used billets for hollow parts. The methods of forming hollow products from hollow semi-finished products include electromagnetic forming, hydroforming, pressure-assisted injection forming, rotary forging, compression, upsetting, rolling, radial extrusion, flanging, upsetting with a controllable deformation zone, and extrusion with a movable sleeve.

In electromagnetic forming, a pulse of current created by discharging a capacitator bank is forced through a work coil that is placed in proximity to the workpiece. The impulse generates a magnetic field around the work coil. This field, in turn, induces eddy currents in the workpiece. This generates an electromagnetic force (the so-called Lorenz force) which causes deformation of the workpiece. In terms of hollow products, this technique is predominantly used for tube bulging and compressing, and it induces only a small change in workpiece wall thickness [1,2]. Yu et al. [3] investigated one-stage and two-stage forming of square tubes from circular tubes. The authors found that the one-stage process was characterized by the occurrence of underfill in the die cavity corners. The use of an additional pre-upsetting operation ensured better flow of the material as well as relatively small changes in the relative wall thickness of the product. Xiong et al. [4] investigated a new process of tube bulging by an attractive electromagnetic force. The main advantage of this method is that it can be used to produce parts with small diameters.

Hydroforming is a metal forming process whereby material deformation is achieved by the utilization of fluid pressure [5]. Forming limit diagrams play an important role in the design of this process [6,7] because they—apart from numerical modeling [8]—facilitate the selection of technological parameters for the process. As a result, it is possible to produce complex geometry parts that are difficult, if not impossible, to fabricate using other methods [9,10]. Applications of this technique for producing hollow products have been studied extensively. Chu et al. [11] proposed a new hydroforming process in which the circumference of the tube was not smaller than the largest circumference of the product. The main advantage of this process is that it can be used to fabricate parts with variable cross-sections using a low-pressure fluid. Xu et al. [12] and Chu et al. [13] combined upsetting and hydroforming, which made it possible to create thin-walled products with only slightly reduced wall thickness.

Pressure-assisted injection forming (PAIF) [14] is a forming method that is similar to upsetting combined with hydroforming. This technique is dedicated to the fabrication of hollow gear shafts [15]. The workpiece is deformed under the load of rigid tools in combination with the action of a pressurizing medium inside the workpiece. Unlike in hydroforming, this medium is not liquid but solid, e.g., polymers, aluminum [16]. Thus, the process does not require using high-pressure hydraulic devices, and the contact between the workpiece and the tools does not have to be very close.

Hollow parts can also be produced by rotary forging. In this process, the outside diameter of the workpiece is reduced by the impact of the dies. Located around the workpiece, the dies perform reciprocating motion in the radial direction and rotary motion about the axis of the workpiece [17]. This process is characterized by high efficiency and makes it possible to manufacture stepped parts of considerable lengths. For this reason, it is very often applied under industrial conditions, as well as it is extensively studied by research centers. Studies investigating rotary forging focus on the effect of selected technological parameters (e.g., process fluctuations [18], the use of a mandrel [19], lubricating conditions [20]) on the stability of the process and product properties. Moreover, rotary forging is also investigated in terms of forging bimetallic products [21,22].

Compression is another method of forming hollow products. In this process, the outside diameter of the workpiece is reduced in selected regions. The process can be conducted with the use of tools performing only translational motion or translational and rotary motion. In the first case, tools have a stepped hole-shaped cavity in which a tube end is placed and deformed [23,24]. In the other case, tools are roller-shaped with a cavity on their surface. Three identical rollers are placed evenly around the workpiece; the rollers rotate about their own symmetry axes and at the same time move towards the workpiece, causing its deformation [25,26,27]. Both techniques make it possible for the process to be conducted in stages, under both hot and cold forming conditions.

Upsetting is used for forming hollow parts with wall thicknesses significantly larger than the thickness of the tube. One can distinguish free upsetting and upsetting in a cylindrical or conical cavity. Parts can be formed with or without the use of a mandrel. Depending on the material grade and part geometry complexity, such parts can be subjected to upsetting under cold, hot, or warm forming conditions. A considerable failure mode in this process is local buckling of the workpiece wall causing overlap and material coherence loss. For this reason, upsetting processes are often performed in several operations between which—in certain cases—an additional heat treatment is applied. Recent studies on upsetting processes for tubes have focused on the design of new upsetting methods or modification of the existing methods to increase their efficiency. For example, Pang et al. [28,29,30] developed a non-isothermal forging process for producing transmission shafts. In this process, the first operation involves heating a tube section which is then subjected to upsetting in a cylindrical die cavity. In effect, this tube section is almost shaped into a bar with a diameter close to the initial outside diameter of the tube. Subsequent stages involve performing upsetting in a conical die cavity and free upsetting. The results confirmed that the proposed method was an effective technique for formation of hollow products with large flanges.

Stepped forged parts can also be produced by rolling methods. Hollow products are often produced by cross wedge rolling and skew rolling. They are formed with the use of tools in the form of plates [31] or rolls [32] with a cavity on their surface, or with the use of rollers that are shaped like truncated cones [33], which makes it possible to achieve the desired profile of parts. However, in many cases, it is also necessary to ensure specified profiles of holes. This is obtained by means of mandrels which are used to improve the manufacturing accuracy of products [34,35]. In addition to that, recent studies on the development of precise rolling methods for hollow products have also focused on the design of numerically controlled rolling mills [36]. Due to their high versatility and efficiency, these machines can be successfully installed in automated production lines.

Another method for forming hollow parts is extrusion. Apart from very widely used forward and backward extrusion processes, radial extrusion is also frequently used. This technique is used to produce parts with flanges, lateral protrusions, etc. Most common failure modes that may occur in this process include a decrease in flange thickness with increasing flange diameter and a risk of material cohesion loss. To prevent the above mentioned phenomena, the process can be conducted with the use of limit rings for constraining free flow of metal in the flange forming zone. Studies have shown that incremental extrusion conducted with the use of both non-deformable rings [37] and deformable rings [38] results in significantly improved quality of extruded parts. The flange has a constant thickness and its maximum diameter is 30% bigger than that obtained in the process conducted without the use of a limit ring.

Inversion is another method of forming hollow parts. In this process, the outside diameter of the tube is increased while the wall thickness of this tube only changes to an insignificant degree. The predominant failure mode occurring in this process is material cracking [39]. Inversion can also be used to produce elements with double walls. In studies [40,41], such products were formed using dies with different fillet radii. Qiu et al. [42,43] formed similar products by free external inversion.

Incremental forming significantly differs from the above-mentioned inversion technique. Incremental forming is conducted using a tool in the form of a mandrel that exerts load on the workpiece and thus causes its local deformation. Thanks to an appropriately designed tool movement trajectory, the degree of deformation increases and apart is gradually formed [44]. This method is quite universal and can be used to form both external [45,46] and internal flanges [47,48], as well as to flange the edges of holes made in metal sheets [49]. In addition to metal forming, incremental manufacturing is increasingly used in additive manufacturing, based on laser forming [50].

Hollow products, the formation of which requires increasing billet wall thickness, can be formed with the use of unconventional methods. These methods include upsetting with a controllable deformation zone and extrusion with a moveable sleeve. In both methods, the workpiece is formed in a closed die cavity which changes its volume during the process. This is made possible by the fact that several tools are moved simultaneously. In the upsetting process, the moving tools are the punch and counter-punch [51], while in extrusion the punch and moveable sleeve [52]. This kinematics of the tools prevent local buckling of the hollow workpiece wall; thus, making it possible to form relatively high flanges with wall thickness larger than that of the billet [53,54]. The process of extrusion with a moveable sleeve can be conducted in several stages, which makes it possible to produce flanges that have a more than twofold higher volume than those produced using the conventional radial extrusion technique [55].

The literature review shows that the existing forming methods can be used to produce forged parts from tubes. These methods significantly differ in terms of process realization, tool design, and kinematics, which means that they are dedicated to the manufacture of specific groups of products. Most of the available forming methods can be used to produce parts with wall thickness similar to that of the tube, whereas only a few methods exist that make it possible to produce stepped parts with varied outer diameters and wall thicknesses. In addition to that, the range of application for individual manufacturing techniques primarily depends on the shape and dimensions of a given part. Parts whose lengths as well as outside and inside diameters in particular regions are close to the dimensions of the tube can be produced in one operation. In contrast, the formation of parts having different dimensions than those of the tube must be conducted in stages, e.g., by sequential upsetting or producing forged parts with the use of several different techniques. The production of forged parts using different methods is a complex process in which an appropriate selection of individual techniques is of vital importance. Due to significant differences between forming methods, only some of them can be combined and used to produce a specific forged part. Consequently, the range of forming techniques available for this purpose is limited. Moreover, the number of studies focusing on the issue of multi-stage forming of parts from tubes with the use of different techniques is limited too.

In light of the above, it was considered reasonable to undertake a study on developing a new forming method for hollow parts. The method is based on extrusion with the use of a moveable sleeve, and parts are formed in a semi-open die cavity. The method is dedicated to manufacturing forged parts directly from hollow [56] or solid billets, or to further processing of stepped forged parts. During extrusion there is a simultaneous increase in the outside diameter and wall thickness of a workpiece region being formed. The study of the proposed method involved performing numerical calculations and experiments, using a hollow stepped shaft (which can be obtained by, e.g., forward extrusion) as a billet. Thus, the proposed method of extrusion with a moveable sleeve was investigated in terms of taking a multi-stage approach to forming hollow parts with the use of different techniques. Results of the study made it possible to determine the effect of basic technological parameters on the stability of the extrusion process and define its failure modes.

## 2. Materials and Methods

**Figure 1:**
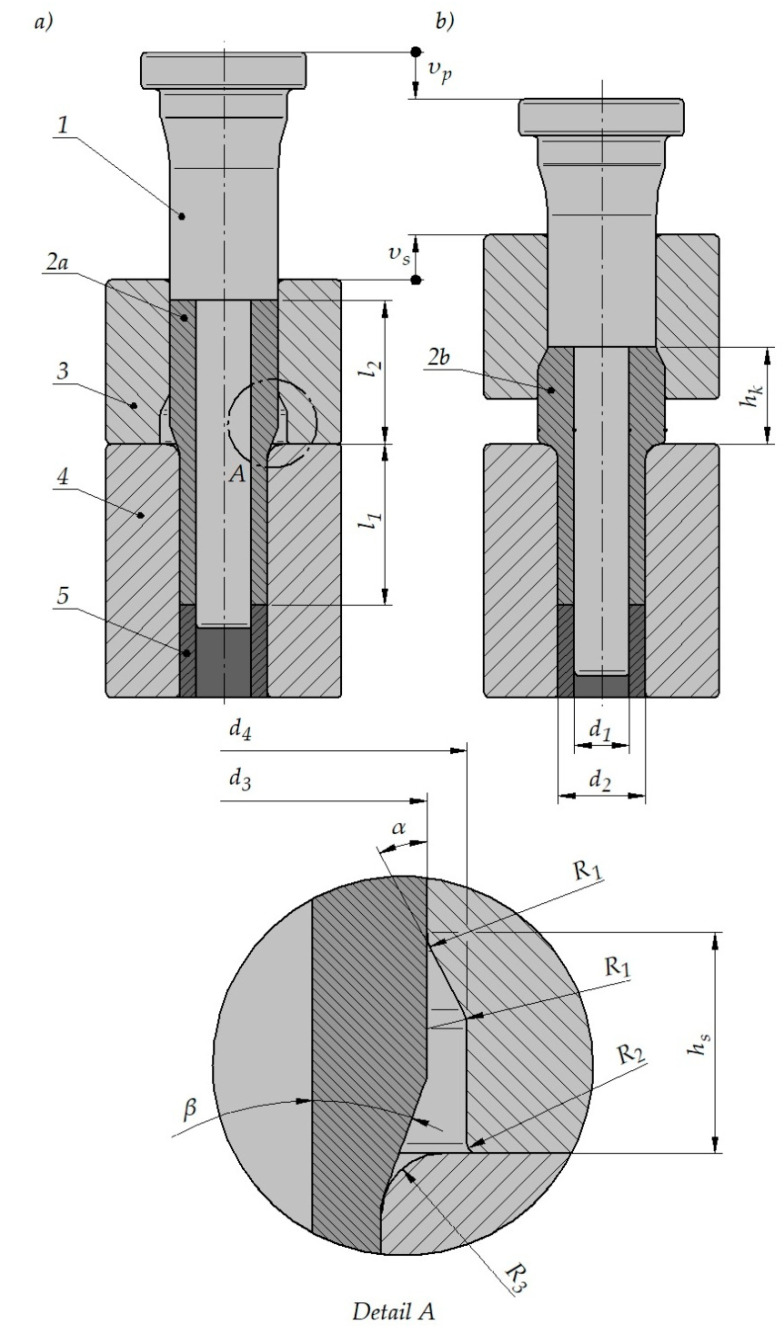
Schematic design of semi-open die extrusion with a movable sleeve; (**a**) start of the process, (**b**) end of the process; 1—punch; 2a—billet; 2b—forged part; 3—movable sleeve; 4—bottom die; 5—ejector.

A schematic design of semi-open die extrusion with a moveable sleeve is shown in Figure 1. In an early phase of the process, the tools consisting of a punch 1, a moveable sleeve 3, a bottom die 4, and an ejector 5 form a closed die, where in a billet 2a is put. The punch 1 moves with a speed υ_p_; thus, filling the die cavity with a height h_s_ made in the moveable sleeve 3 with the material. After that, the sleeve 3 begins to move with a speed υ_s_ in the opposite direction to that of the punch 1. As a result, the initially closed die formed by the tools opens up and increases its volume. In effect, a successive phase of the extrusion process takes place in a semi-open die, which makes it possible to increase the height of the increased outside diameter region on the forged part 2b to the final value h_k_.

It was assumed that semi-open die extrusion with a moveable sleeve would be performed as a second operation in the forming process of stepped hollow parts. Consequently, the billet was modeled as a shaft with a constant diameter hole and two increased outside diameter are as that could be produced, e.g., by forward extrusion (Figure 1a).The process of semi-open die extrusion with a moveable sleeve was used to increase the diameter and wall thickness of the increased diameter region on the workpiece, with the cross-sectional dimensions of the smaller diameter region remaining constant.

Different dimensions of the billet and tools were tested in order to determine their effect on the extrusion process. The following were variable: a diameter d_4_ and a height h_s_ of the moveable sleeve cavity, a chamfer angle β between increased outside diameter areas on the workpiece and a chamfer angle α in the sleeve cavity. Other dimensions remained constant. A detailed list of the analyzed cases is provided in Table 1. In every tested case, the punch speed was maintained constant at υ_p_ = 5 mm/s. The moveable sleeve speed depends on the punch speed as well as tool and billet dimensions. This speed can be calculated from the constant volume principle. The volume of a material extruded per unit of time from the die using a moveable sleeve with a diameter d_3_ must be equal to an increment in the volume (resulting from sleeve motion) of the cavity that is formed in the zone between the diameters d_3_ and d_4_ of the moveable sleeve. The speed υ_s_ of the moveable sleeve was calculated in compliance with denotations given in Figure 1 using Equation (1).

(1) $$\upsilon_{s}=\upsilon_{p}\cdot \frac{d_{3}^{2}-d_{1}^{2}}{d_{4}^{2}-d_{3}^{2}}.,$$

**Figure 2:**
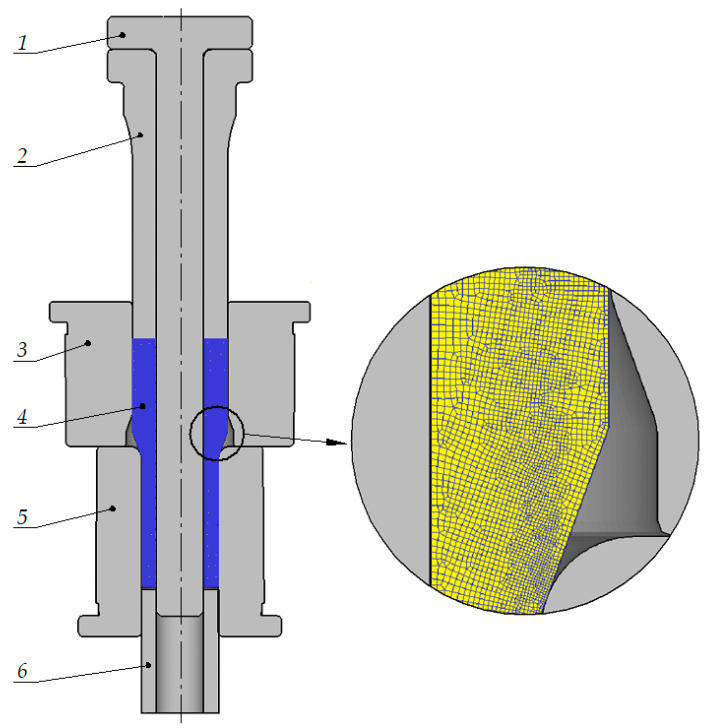
FEM model of semi-open die extrusion with a movable sleeve; 1—mandrel; 2—punch; 3—movable sleeve; 4—billet; 5—bottom die; 6—ejector.

Numerical simulations of the analyzed extrusion process were performed using the finite element method in Deform-2D/3D. An FEM model of the analyzed process is shown in Figure 2. The numerical model is the same as the device for experimental tests. The boundary conditions for the simulation are as follows. The simulations were performed using an axisymmetric geometry type. The extrusion process was conducted under cold working conditions, and the temperature of the tools, billet, and environment was set to 20 °C. The tested material (both in the numerical analyses and experiments) was aluminum alloy EN AW 6060 in an annealed state, with its flow curve described by a constitutive Equation (2) [37]. The flow curve was determined at a temperature of 20 °C (the same temperature as the extrusion process was carried out) for three different strain rates, the ratio of which was 1:10:100. Due to the value of the flow stress for individual strain rates being almost the same, the flow curve was described by an equation dependent only on the strain. The flow curve for higher temperatures (and different strain rates in this temperatures) was taken from the database of the Deform-2D/3D program. The billet was modeled as a plastic object while the tools were modeled as rigid objects. The billet was discretized using two-dimensional tetragonal, four-node elements. Contact between the workpiece and the tools was described by the shear friction model, with a friction factor of m = 0.2; the workpiece-tool heat transfer coefficient was set to 12 kW/m^2^K [37]. The friction conditions were tested by upsetting ring specimens between flat dies. The samples were upset to about half the initial height. The value of the friction factor was determined by comparing the dimensions of the samples obtained in the experiment with the dimensions of the samples obtained in numerical simulations.

(2) $$\sigma_{p}=147.5\;\cdot \;\varepsilon^{0.2},$$

where *σ_p_* is the flow stress, *ε* is the strain.

**Figure 3:**
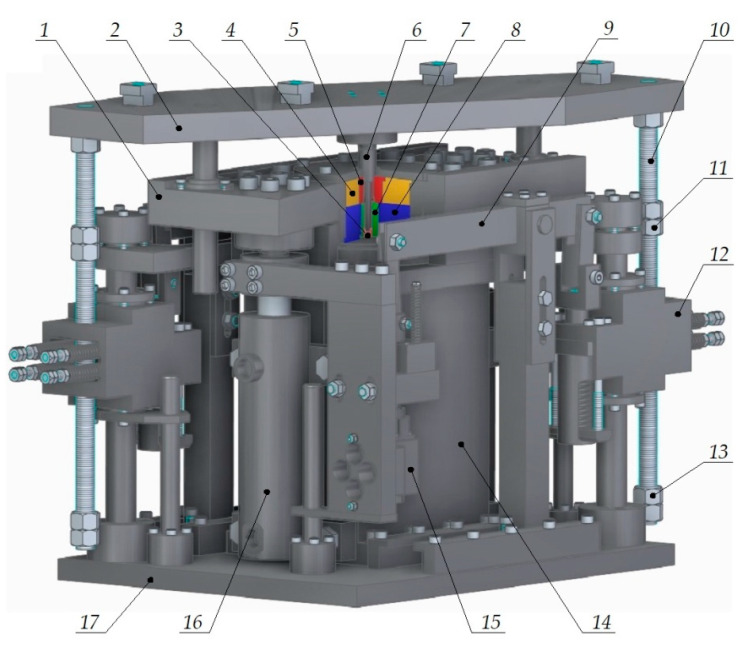
A 3D model of a device for semi-open die extrusion by moveable sleeve; 1—middle plate; 2—top plate; 3—ejector; 4—shrink ring of moveable sleeve; 5—moveable sleeve; 6—punch; 7—bottom die; 8—shrink ring of bottom die; 9—lever; 10—screw; 11—top nut; 12—lever power unit; 13—bottom nut; 14—base; 15—manipulator; 16—servomotor; 17—bottom plate.

**Figure 4:**
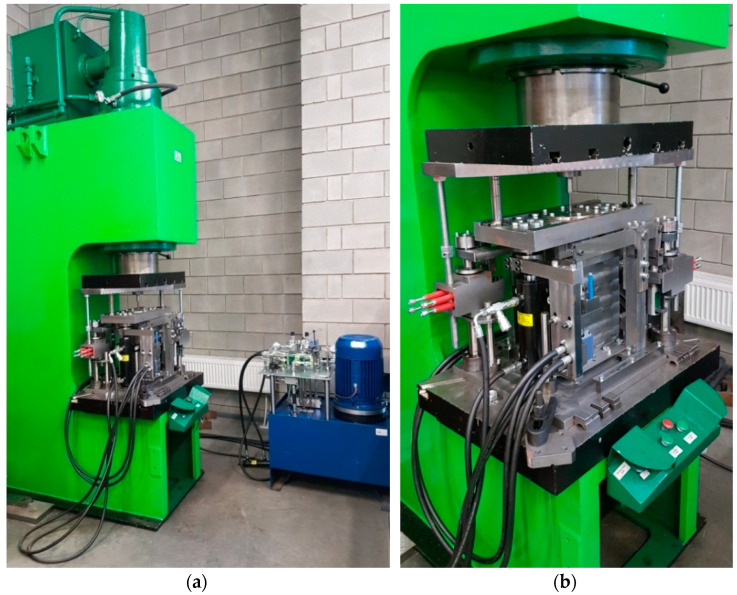
Test stand for performing semi-open die extrusion with a moveable sleeve: (**a**) PYE 160SS hydraulic press provided with a hydraulically driven extruding device, (**b**) view of the working space of the hydraulic press.

Experiments were conducted with the same parameters as those applied in the numerical analysis. The experiments were conducted under laboratory conditions on a test stand shown in Figure 3 and Figure 4. The test stand consists of a device for semi-open die extrusion using a moveable sleeve, with the subassemblies of this device being driven by a hydraulic press (PYE 160SS) and hydraulic feed system.

The design and operating principle of the extruding device are described based on its three-dimensional model shown in Figure 3. To the hydraulic press slide a top plate 2 is fixed, in which punch 6 and screws 10 are mounted with top nuts 11 bottom nuts 13. A moveable sleeve 5 and its shrink ring 4 are mounted to a middle plate 1, and a bottom die 7 (pre-stressed by means of a shrink ring 8) is fixed to a base 14. In the hole of the bottom die 7 is an ejector 3 which is connected to the hydraulic press ejector. The base 14 is fixed to a bottom plate 17 which is mounted on the hydraulic press table. On the base 14 hydraulic servomotors 16 are mounted, the pistons of which are fixed to the middle plate 1. To the servomotor pistons hydraulic manipulators 15 are fixed, the slides of which are connected by means of a variable leverage lever 9 to a lever power unit 12. The manipulators 15 are connected to a hydraulic feed system provided with an electrically driven gear hydraulic pump.

The press slide drives the top plate 2, causing motion of the punch 6 and screws 10 simultaneously. As a result of the load exerted by the top nuts 11 on the lever power unit 12, a slide of the hydraulic manipulator 15 begins to move; thus, causing pistons of hydraulic servomotors 16 to move. As a result, the moveable sleeve 5 starts to move, which makes it possible to run the extrusion process in compliance with the description above. For calibrating the extruding device only two parameters must be adjusted appropriately. With the appropriate leverage of the lever 9, the required speed ratio between the punch 6 and the moveable sleeve 5 is achieved. Movement of the punch 6 that sets the moveable sleeve 5 in motion is controlled by adjusting the location of nuts 11 relative to screws 10.

## 3. Results and Discussion

**Figure 5:**
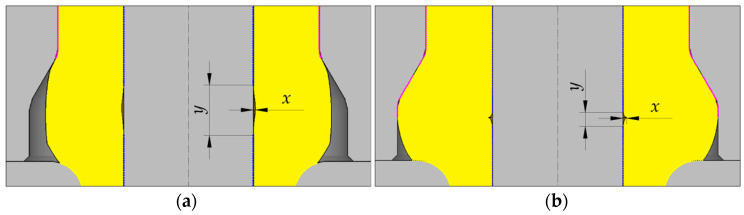
No-contact zones between the workpiece and the mandrel in selected stages of an early phase of semi-open die extrusion with a moveable sleeve: (**a**) beginning of the process, (**b**) moment of first contact of the material with the movable sleeve impression with a diameter d_4_.

The first step in the analysis of the semi-open die extrusion process with a moveable sleeve was to determine the effect of selected parameters on the stability in an early phase of the extrusion process. In this phase, the sleeve does not move and the closed die cavity described by a height h_s_ is filled with the material. One failure mode that may occur in this phase of the extrusion process is a loss of contact between the workpiece and the mandrel when the extruded flange reaches the target diameter d_4_ (Figure 5b). Still, the workpiece may lose contact with the mandrel well before the flange achieves the assumed diameter, right after starting motion of the punch (Figure 5a). Depending on selected technological parameters of the extrusion process, the underfill can either be removed before the flange reaches the target diameter d_4_ (meaning that the process parameters are correct) or not. To establish a relationship between the analyzed parameters and underfill, the *δ* parameter described by Equation (3) was determined. The degree of underfill was estimated based on the value of *δ*—the higher the value of the *δ* parameter is, the greater the underfill is and the more difficult it is to remove it.

(3) $$\delta =\frac{x}{y},$$

where *x* is the greatest radial distance between the workpiece wall and the mandrel (see Figure 5a), or the distance when the flange has achieved the target diameter (see Figure 5b), *y* is the length (in axial direction) of the no-contact zone between the workpiece and the mandrel when the *x* distance is the highest (see Figure 5a), or the distance when the flange achieves the target diameter (see Figure 5b).

**Figure 6:**
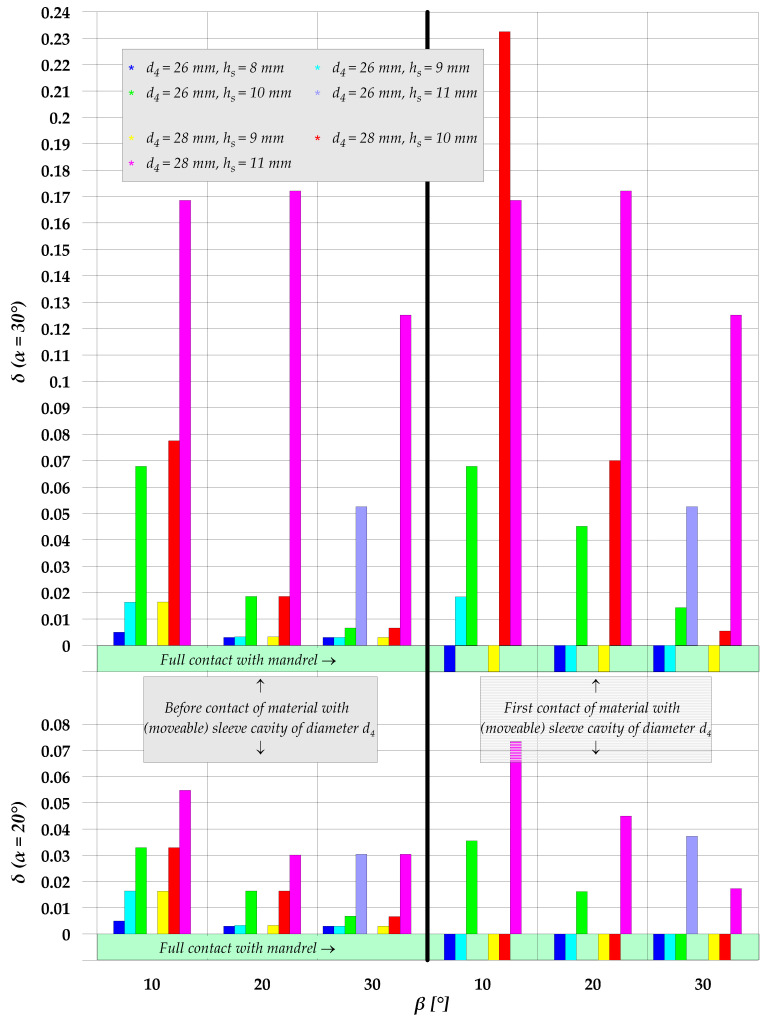
Parameter *δ* for the analyzed cases of semi-open die extrusion with a moveable sleeve.

The *δ* parameter was determined for two selected stages in an early phase of the extrusion process. The first stage was the moment when the radial distance between the workpiece wall and the mandrel was the greatest, while the other was the moment when the extruded flange reached the target diameter d_4_. The *δ* parameter values obtained for all analyzed cases of the extrusion process are given in Figure 6. For most cases, the first stage (i.e., maximum radial distance between the workpiece wall and the mandrel) takes place when the diameter of the flange is smaller than the target value, i.e., prior to contact between the workpiece and the moveable sleeve cavity with a diameter d_4_. For these cases of the extrusion process, two different situations can be observed. In the first one, the underfill is removed before the flange reaches the target diameter. This is tantamount to full contact between the workpiece and the mandrel, and it means that the parameters were selected correctly. In the other situation the underfill is not removed; therefore, the flanged product with the target diameter has a defect in the form of underfill. In the second stage of an early phase of the extrusion process (i.e., the flange has the target diameter), three different situations can be distinguished with respect to the *δ* parameter value. In the first situation, the product is correct (no underfill is observed). In the second one, the underfill is smaller than that occurring before the target flange diameter is reached. As for the third situation, the underfill developed after starting the punch motion is the greatest. An analysis of the relationship between the investigated parameters of extrusion and the *δ* parameter has shown that increase in both h_s_ and β leads to increased underfill. Regarding the chamfer angle α in the moveable sleeve cavity, it can be observed that for the cavity height h_s_ equal to 8 and 9 mm the α angle value has no effect on the *δ* parameter. Nevertheless, for higher values of h_s_ an increase in the α angle leads to an increase in the degree of underfill described by the *δ* parameter. The effect of the flange diameter d_4_ on the *δ* parameter value is significant for the cases of extrusion conducted with the chamfer angle set to α = 20°. For these cases, the flange diameter increase causes a decrease in the *δ* parameter when the flange reaches the target diameter d_4_. 

**Figure 7:**
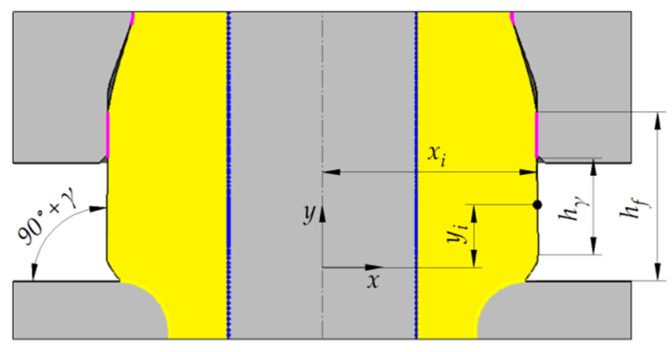
Forged part with limit flange heights. The schematic also shows workpiece/tool contact zones.

**Figure 8:**
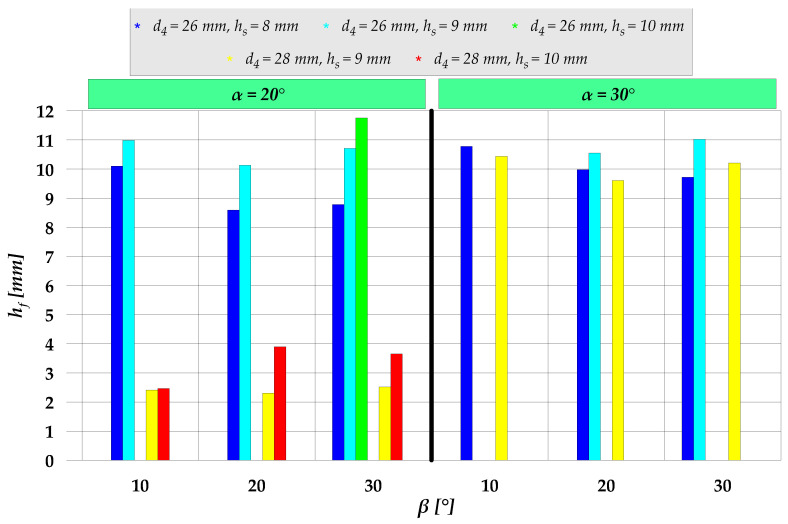
Limit values of the flange height h_f_ obtained for the analyzed cases of semi-open die extrusion with a moveable sleeve.

Based on the above analysis, it was possible to determine extrusion cases in which an early stage of this process is stable, i.e., the material completely fills the hole in the forged part when the flange reaches the target diameter d_4_. In Figure 6, these cases are marked as *full contact with mandrel*. Further analyses were conducted for these cases of the extrusion process. When the target diameter of the flange is reached, the sleeve is set in motion in the opposite direction to that of the punch. In effect, the flange height is increased. This height increase is however limited. During the extrusion process the workpiece loses contact with the mandrel in the flange forming zone. In light of the above, the maximum flange height at which the workpiece is in full contact with the mandrel was taken to be the limit flange height h_f_ (Figure 7). Limit flange values obtained for the analyzed cases of the extrusion process are provided in Figure 8. It should be mentioned, however, that in spite of the fact that the workpiece and the mandrel are no longer in contact, the extrusion process can be continued (in order to increase the flange height), because the maximum radial distance between the workpiece and the mandrel is small and increases as the extrusion process progresses. Therefore, the allowable distance depends on the dimensional tolerance for a hole in the forged part.

The obtained values of the limit flange height h_f_ demonstrate that the smallest flange height is obtained when the extrusion process is conducted using a chamfer angle of α = 20° for a flange diameter of d_4_ = 28 mm. For this case, there is no workpiece/mandrel contact after starting the sleeve motion. Consequently, the sleeve motion does not lead to an increased flange height, which means that the process parameters were incorrect. As far as the other cases are concerned, the extrusion process runs in a stable way, with the sleeve motion causing an increase in the flange height. It can be observed that an increase in the sleeve cavity height h_s_ and the chamfer angle α leads to an increase in the limit flange height h_f_. On the other hand, an increase in the diameter d_4_ results in a reduced limit flange height. It is, however, difficult to determine a clear relationship between the chamfer β angle value and the feasible flange height. Still, for the analyzed cases, the highest flange height values were predominantly obtained for the smallest value of the β angle.

Forged parts with limit height flanges are characterized by variable flange diameter. The diameter is the highest and at the same time greater than the target diameter value d_4_ in proximity of the bottom die. In contrast, the closer it is to the moveable sleeve, this diameter becomes smaller and closer to the target value. In effect, the generating line of flange flank for h_γ_ is similar to the section inclined away from the symmetry axis of the workpiece by an angle *γ* (see Figure 7). Therefore, the generating line of flange flank for the h_γ_ dimension is a conical surface. Values of the *γ* angle for the analyzed cases of extrusion (except for the cases described by α = 20°, d_4_ = 28 mm, in which the flange height increase is incorrect) were determined by measuring the flange radius for the h_γ_ dimension. Results were saved as the coefficients of measuring points x_i_ and y_i_ relative to the adopted reference system (see Figure 7). The *γ* angle was calculated using linear regression. The measuring points were fitted with a straight line described by Equation (4), whereas the *γ* angle was calculated from Equation (5).

(4) y(x)=a·x+b,

(5) γ=arctg (a)−90°,

where *a* is the slope of a straight line, *b* is the free term.

**Figure 9:**
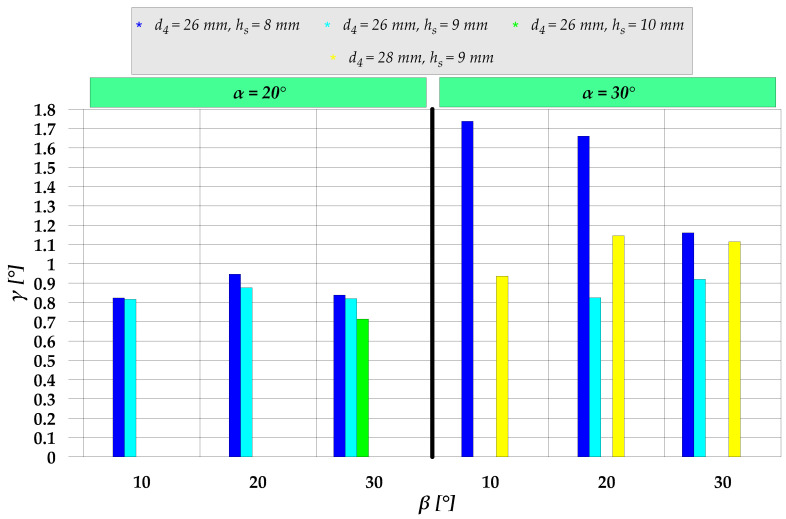
Values of the flange flank inclination angle *γ* for the analyzed cases of semi-open die extrusion with a moveable sleeve.

Calculated values of the *γ* angle are given in Figure 9. An increase in the height h_s_ leads to a decrease in the *γ* angle value. The greatest difference in this respect can be observed for the flanges extruded using the following parameters: d_4_= 26 mm, h_s_= 8 mm, α= 30°, β= 20°and 30°. In contrast, an increase in the diameter d_4_ causes an increase in the *γ* angle. A similar situation can be observed when the chamfer angle α in the sleeve cavity is increased.

**Figure 10:**
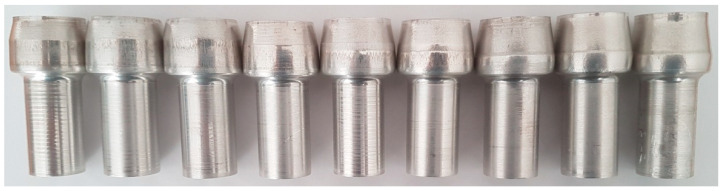
Forged parts obtained from experiments.

**Figure 11:**
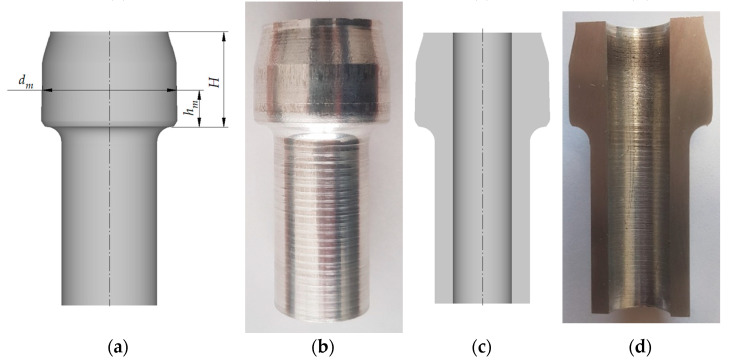
View of a forged part obtained from:(**a**) numerical analysis, (**b**) experiment; cross section of a forged part obtained from:(**c**) numerical analysis, (**d**) experiment.

Experiments were performed for the extrusion process conducted with the following parameters: d_4_ = 26 mm, h_s_ = 9 mm, α = 20°, β = 20°, l_2_ = 26 mm. In this process, the forged part has a flange with a height bigger than the assumed limit height. Forged parts obtained from the experiments are shown in Figure 10, while Figure 11 shows forged parts obtained from the numerical analysis and experiments.

Forged parts obtained from the numerical analysis and experiments were then compared in terms of flange diameters. Measurements were made at different flange heights, as shown in Figure 11a. Results are provided in Table 2. An analysis of the results demonstrates that the highest agreement was obtained for the h_m_ values up to 11 mm and above 18 mm. The greatest difference between the diameters is 0.07 mm, while the lowest is 0.02 mm. For the h_m_ values ranging between 12 and 17 mm, the discrepancies are slightly larger and amount up to 0.18 mm. During extrusion, this region of the workpiece is inside the sleeve cavity at a height of the chamfer described by the α angle. This region of the workpiece has no contact with the sleeve; therefore, radial flow of the material is unconstrained, which can explain the higher dimensional discrepancies. Compatibility of the numerical calculations with the results of experimental tests was also completed on the basis of the additional analysis of the dimensions of the forgings. For this purpose, the value of the standard deviation was determined for the value of the flange diameter d_m_ within the h_m_ dimension range 3÷11 mm. In this zone, the diameter of the flange has a similar value along its entire height. The value of the standard deviation determined for the dimensions of the forgings obtained from experimental tests and numerical simulations is 0.1 mm. This proves the good compliance of the numerical model with the real object, which is also confirmed by the small difference between the *γ* angle values for the analyzed cases amounting to 0.008°. Moreover, the relatively small value of the standard deviation shows that the diameter of the flange is close to the average value over the entire height. In other words, this means that the flange side surface is close to the cylindrical surface—as expected.

The experimental results demonstrate that, apart from the above-analyzed parameters (d_4_, α, β, h_s_), the configuration of the extruding device has a significant impact on the extrusion process too. In an early stage of the extrusion process when the moveable sleeve remains motionless, the moment when this stage ends is of key importance. It is tantamount to the moment when the lever power unit is set in motion (item 12 in Figure 3). If the sleeve begins to move too early, underfill occurs; on the other hand, if the sleeve begins to move too late, this will cause overfill and tool overload. Bearing the above in mind, the extruding device must be calibrated in such a way that the moment of starting the sleeve motion is gradually delayed. In effect, the risk of tool overload is very low. After setting the sleeve in motion, the speed of the sleeve, which is adjusted by leverage (item 9 in Figure 3), is the most important parameter. If the speed is too high relative to the speed of the punch, underfill occurs. As a result, the formed flange has a smaller diameter than required, while the hole diameter in the flange region is bigger than the diameter of the mandrel. Thus, a too high speed of the movable sleeve leads to an increased radial distance between the workpiece wall and the mandrel and at the same time causes a reduced increase in the workpiece wall thickness. If the sleeve speed is too low, the opposite is observed. Overfill occurs, which leads to tool overload and obtaining a flange diameter that is bigger than required. 

Another important technological parameter is the moment when the extrusion process ends. The experimental results demonstrate that the bottom slide settings were correct. If the press is stopped too late, this leads to axial compression of the forged part. As a result, the forged part is damaged because the hole diameter is increased (the wall of the workpiece loses contact with the mandrel). The results reveal that variations in the press speed (e.g., temporary deceleration due to load increase or variable slide speed due to manual operation of the press) have no significant impact on the process stability. This results from the fact that the sleeve’s speed depends on the speed of the punch. Due to the use of the mechanical hydraulic closed system for sleeve speed control, the speed of the sleeve changes with the punch speed. A key parameter of the analyzed extrusion process is a ratio between these two speeds—its value must remain constant during extrusion and be dependent only on leverage (item 9 in Figure 3).

## 4. Conclusions

The results of the study investigating semi-open die extrusion with a movable sleeve led to the following conclusions:

- The extrusion process has two distinct stages: the first one takes place before the moveable sleeve is put into motion, and the other describes the time when the sleeve is moving in the opposite direction to that of the punch.
- As for the first stage of the extrusion process, the primary failure mode is underfill in the forged part hole; the occurrence of this failure mode primarily depends on the cavity height h_s_ and the chamfer angle β between the regions with different outside diameters—the risk of underfill increases with increasing these parameters.
- Main failure modes in the other stage of the extrusion process concern the maximum achievable flange height and flange flank inclination.
- An increase in the sleeve cavity height h_s_ and the chamfer angle α in this cavity leads to an increase in the maximum feasible flange height, whereas an increase in the flange diameter d_4_ leads to a decrease in this height.
- An increase in the cavity height h_s_ results in a decrease in the flange flank inclination angle, whereas an increase in the diameter d_4_ and the chamfer angle α leads to an increase in the inclination angle of the flange flank.
- For the extrusion process to be stable, it is necessary to ensure correct configuration of the extruding device, the most important parameters being the moment of starting the sleeve motion, punch/sleeve speed ratio, and the end of the extrusion process.

## Figures and Tables

**Table 1:** List of the analyzed cases of semi-open die extrusion with a moveable sleeve (denotations in the table are the same as those used in Figure 1).

**d_1_** (mm)	**d_2_** (mm)	**d_3_** (mm)	**d_4_** (mm)	**R_1_** (mm)	**R_2_** (mm)	**R_3_** (mm)
11.5	18.5	23	26; 28	2	0.5	3
**l_1_** (mm)	**l_2_** (mm)	**α** (°)	**β** (°)	**h_s_** (mm)	**υ_p_** (mm/s)	**υ_s_** (mm/s)
34	60	20; 30	10; 20; 30	8; 9; 10; 11	5	acc. to Equation (1)

**Table 2:** Dimensions of forged parts obtained from numerical analyses and experiments (denotations in the table are provided in accordance with those in Figure 11).

h_m_ (mm)	d_m_ (mm) Experiment	d_m_ (mm) FEM	H (mm) Experiment	H (mm) FEM	SD_dm-E_^1^ (mm)	SD_dm-F_^2^ (mm)
3.00	26.37	26.32	18.5	18.46	0.1	0.1
4.00	26.32	26.26				
5.00	26.27	26.20				
6.00	26.21	26.14				
7.00	26.17	26.10				
8.00	26.13	26.06				
9.00	26.06	26.01				
10.00	26.03	26.00				
11.00	26.02	26.00				
12.00	25.85	26.00				
13.00	25.56	25.74				
14.00	25.08	25.24				
15.00	24.52	24.66				
16.00	23.90	24.08				
17.00	23.30	23.44				
18.00	23.03	23.00				
18.46	23.03	23.00				

^1^SD_dm-E_—standard deviation of dimension d_m_ obtained in experiment, for h_m_ = 3 ÷ 11 mm. ^2^SD_dm-F_—standard deviation of dimension d_m_ obtained in FEM, for h_m_ = 3 ÷ 11 mm.

## Data Availability

Data is contained within the article.
